# Relaxation or Regulation: The Acute Effect of Mind-Body Exercise on Heart Rate Variability and Subjective State in Experienced Qi Gong Practitioners

**DOI:** 10.1155/2021/6673190

**Published:** 2021-06-08

**Authors:** Florens Goldbeck, Ye Lei Xie, Martin Hautzinger, Andreas J. Fallgatter, Gorden Sudeck, Ann-Christine Ehlis

**Affiliations:** ^1^Lab for Psychophysiology and Optical Imaging, Department of Psychiatry and Psychotherapy, University Hospital Tübingen, Calwerstraβe 14, Tübingen 72076, Germany; ^2^Department for Traditional Chinese Sports, Shanghai University of Sport, Changhai Road 399, Shanghai 200438, China; ^3^Department of Psychology, Division of Clinical Psychology and Psychotherapy, Eberhard-Karls University Tübingen, Schleichstraße 4, Tübingen 72076, Germany; ^4^LEAD Graduate School & Research Network, University of Tübingen, Gartenstraße 29, Tübingen 72074, Germany; ^5^Institute of Sports Science, Department of Education & Health Research, Eberhard-Karls University Tübingen, Wächterstraβe 67, Tübingen 72074, Germany

## Abstract

Mind-body exercises such as Yoga or Qi Gong have demonstrated a wide range of health benefits and hold great promise for employment in clinical practice. However, the psychophysiological mechanism underlying these effects remains unclear. Theoretical frameworks highlight *regulation* as a characteristic and specific mechanism of mind-body exercise for which empirical evidence is scarce. To investigate the exact nature of this mechanism, we tracked acute changes in autonomic nervous system (ANS) activity and subjective state over a common form of mind-body exercise (Qi Gong). Heart rate variability (HRV) and subjective state were assessed in 42 Qi Gong practitioners from China and Germany during a standard moving Qi Gong exercise (Baduanjin). *Relaxation* in supine position prior and after the exercise served as a control condition to Qi Gong and to assess changes before and after the exercise. Following Qi Gong, all practitioners reported significantly increased subjective calmness and perceived body activation, attentional focus, and subjective vitality. On the physiological level, a significant decrease of parasympathetic modulation and increase in heart rate indicated a pattern of moderate general physiological activation during Qi Gong. A significant increase in overall RR-interval modulation and cardiac coherence during Qi Gong were indicative of a mechanism of active regulation. Examination of the RR-interval trajectories revealed a rhythmic pattern of ANS activation and deactivation in sync with activating and relaxing segments of the exercise. Significant changes in subjective state, not on the physiological level, before and after the exercise were observed. Significant associations between Qi-Gong-specific beliefs, age, cultural background, and experiential and physiological measures demonstrated the complexity of mind-body exercises as multicomponent interventions. Overall, this study highlights moderate general physiological activation, exercise-dependent rhythmic ANS modulation, and induction of a characteristic state of eutonic calmness as potential psychophysiological mechanisms underlying the health benefits of mind-body exercise.

## 1. Introduction

Mind-body exercise [1], mind-body therapies [2], or meditative movement [3, 4] describe a group of practices characterized by a combined focus on movement/posture, patterns of breathing, and mental activity. Over the last decades, mind-body exercises, such as Yoga, Taijiquan, or Qi Gong, have become a global phenomenon in the pursuit of private and public health endeavors [5, 6]. A growing number of studies have therefore started to investigate their effects on the level of health outcomes; however, the exact mechanism on which these effects are based remains unclear.

One prominent example from the group of mind-body exercises is Qi Gong (Chinese: 气 Qi for vital energy; 工Gong for work, cultivation). Like other mind-body practices, Qi Gong consists of a multitude of forms and styles, practiced for various purposes [7]. Baduanjin (“Eight pieces of brocade”) is a form of moving, health-cultivating Qi Gong, which will be used as a representative mind-body exercise in this article because a standard form of this traditional Qi Gong exercise has been developed and increasingly used in research [8]. Qi Gong in the following sections refers to the moving, standard forms of health-cultivating Qi Gong.

Qi Gong has shown promising effects in the alleviation of some of society's most debilitating conditions [9–13]. Risk factors such as stress [14] and sleep quality [15] are responsive to regular Qi Gong practice, and evidence about its long-term benefits on a physiological level is beginning to accumulate [2, 11, 16–18]. However, equivocal evidence still exists regarding the acute physiological and subjective experiential effect of Qi Gong–a gap we aimed to address in this study.

*Relaxation* is a term often used to describe the impact of mind-body exercise on stress and symptomatology [3, 10, 17]. However, the concept of relaxation conveys ambiguity with regard to ANS activity and practitioners' subjective state [4]. It refers to a state of “hypotonic slackness” (e.g., dozing off on the couch in a state of limpness) as well as a state of “eutonic calmness” (e.g., expecting the starting signal for a 50 m sprint, mentally calm with optimal muscular tone) [4]. Payne and Crane-Godreau [4] define eutonic calmness, a state of balanced muscular tone (eutonic) and mental calmness, as the characteristic state of mind-body exercise [3, 4]. The difference between hypotonic slackness and eutonic calmness in subjective state presumably corresponds with a difference on the level of ANS configuration (parasympathetic dominance vs. parasympathetic/sympathetic balance) and long-term effects on the level of health outcomes.

According to Qi Gong theory [7], eutonic calmness follows from the active alignment of posture/movement, breath, and mental activity during practice, a process we summarize under the term, *psychophysiological regulation.* Regulation in this sense refers to an active process in the practitioner, which is different from passive relaxation. Some descriptive works have highlighted regulation in mind-body exercise as a means to achieve mental and physiological homeostasis [2, 4, 16]. However, to our knowledge, no study has directly tested regulation vs. relaxation as acute mechanism of mind-body exercise. To do so, we tracked acute changes in practitioner's subjective experience and ANS activity over the course of relaxed resting condition and the practice of a standard moving Qi Gong exercise (Baduanjin). To capture the specific state induced via the practice of Qi Gong [3, 4, 7], changes on an experiential level were assessed using self-report dimensions linked to eutonic calmness (e.g., calmness, perceived body activation), regulation (subjective vitality), and complementary concepts from the area of body-mind exercise (e.g., sensation of Qi). To measure the acute influence of Qi Gong on ANS activity, we used standard and specific parameters of HRV [19].

One prominent indicator reflecting regulation via psychological and physiological influences in the ANS is HRV, the variability of adjacent heart beat intervals [20]. Reduced HRV has been linked to health impairment and associated risk factors [21–23]. Concerning Qi Gong's influence on HRV, the majority of studies has looked at changes of resting state HRV in Qi Gong beginners following training protocols of 12–24 weeks [24–29]. Findings in these studies have been equivocal.

Regarding acute changes of HRV parameters, Lin, Wei [30] found no acute changes in the low frequency (LF) and high-frequency power following 18 Forms Tai Chi International Qi Gong. However, the authors reported a significant decrease in the LF-spectrum and increase in the HF-spectrum towards the end of the practice. Results of the acute influence of Baduanjin, the Qi Gong form used in the present study, on HRV are not known. To measure the acute influence of Qi Gong on HRV, we assessed standard HRV parameters in the time and frequency domain [19, 20] before, during, and following two bouts of Baduanjin. A specific parameter associated with increased well-being and psychophysiological balance is cardiac coherence [31–33]. Earlier findings mentioned Qi Gong as a promising candidate to induce a state of coherence [32]; however, no study has empirically tested this assumption. To assess Qi Gong's quality as a mind-body exercise, we included cardiac coherence as a potential correlate for the specific state achieved through the adjustment of mind, body, and breath in Qi Gong [7].

Qi Gong directly translates as vital energy cultivation. In Western psychology, vitality on an experiential level has been adopted as subjective vitality (SV), “a dynamic aspect of well-being marked by the subjective experience of energy and aliveness” [34, 35]. Subjective vitality has been linked to regulative capacity of the self [36] and described as distinct from relaxed “nonactivated states such as happiness, satisfaction, and contentment” [37, 38]. Hence, subjective vitality was used as an integrated measure to capture the state of eutonic calmness [4] associated with optimal regulation during Qi Gong. To characterize the state in more detail, we complimented the measure of SV with subcomponents relevant to the distinction between hypotonic relaxation and eutonic calmness [4]. Thayer [39] used the term *calm energy* to describe a state simultaneously high in calmness and in energetic arousal in a two-dimensional space. To mirror these aspects, we assessed perceived calmness and body activation in the present study. Based on Larkey's [3] definition of Qi Gong as meditative movement, we expected further state characteristics of increase in attentional focus, a heightened body awareness, and an increase in pleasant body sensations.

A unique feature of the common mind-body exercises (i.e., Yoga, Taiji, and Qi Gong) is the pervasive influence of their respective cultural background (language, beliefs, rituals, practice habits, assumed stages of development, etc.). In comparison to other forms of exercise, this makes body-mind exercise a multicomponent intervention with inherent challenges to the examination within a classical randomized control trial design [40, 41]. To approach this problem from a different angle, we applied a within subject design and explored the influence of four covariates potentially relevant to the acute effect of Qi Gong: To examine the influence of cultural background, we recruited participants from an incongruent (Germany) and a congruent cultural background (China) and reexamined overall effects in the two national subsamples. For the influence of Qi-Gong-specific beliefs, participants filled out a Qi-Gong-specific self-report measure (method section) developed for this study. Different levels of mastery in the respective exercise are assumed to enable different types of inner experiences and physiological response in practitioners [7, 42]. Hence, we recruited practitioners with varying amounts of experience and used “years of Qi Gong experience” as a covariate. Age was used as a covariate due to the predominance of interest in Qi Gong among older populations and the demonstrated influence of age on HRV [20]. In applying these covariates and the respective design, we aimed to complement findings from randomized control trials and detangle global from specific effects characteristic for the practice of Qi Gong.

The overall goal of this study was to explore the psychophysiological mechanism underlying mind-body exercise via the examination of acute changes in subjective state, HRV during rest, and a standard moving Qi Gong exercise. We assumed that Qi Gong would increase levels of subjective vitality and related subjective indicators to a state of increased perceived body activation and calmness, with a concurrent high degree of body awareness and attentional focus that would feel pleasurable. Based on the equivocal results of the literature, an exploratory approach was used with regard to the impact of Qi Gong on HRV parameters. The covariates' cultural background, beliefs, experience, and age were controlled in order to gain a better understanding of potentially underlying mechanisms in the acute effects of Qi Gong practice.

## 2. Material and Methods

### 2.1. Sample

Qi Gong practitioners were recruited in mainland China (*n* = 21) and in Germany (*n* = 21). The Chinese group consisted of former and temporary students at the Shanghai University of Sport. The German sample consisted of amateur practitioners and coaches from various Qi Gong training locations in south Germany. The bi-national protocol was approved by the “Ethics Committee at the University Hospital and University of Tübingen” and all subjects gave written informed consent in their respective mother language in accordance with the Declaration of Helsinki. The inclusion criteria were: free of current medication, no cardiovascular disease or diabetes, and ability to perform the Qi Gong exercise Baduanjin. The basic characteristics of the overall sample and the two national subgroups are described in Table 1 and Additional file 1.

### 2.2. Measures

#### 2.2.1. Subjective State

The study aimed to measure a specific configuration in subjective state following regulation during Qi Gong. Seven items were selected, partly from larger measures, to capture the full state and configuration of distinct subdimensions of eutonic calmness. Indicator items were used for means of time efficacy:

Subjective vitality was employed as the main measure to capture the state of eutonic calmness. Two items from the subjective vitality scale [34] were used as indicator items (“I feel vital and alive;” “I have energy and spirit”). The items were framed as “At the moment.” The average inter-item correlation in this study was *r*_*M*_ = 0.87. To validate the effect of Qi Gong from a complementary theoretical perspective, we included one item in a Qi Gong specific wording ([43]; “I can feel my Qi”), which was also supposed to resemble the full state of eutonic calmness.

To characterize a specific configuration of state via its subdimensions, we included two items mirroring Thayer's [39] theory of calm energy (“I feel calm;” “My body feels activated”). The theory assumes a two-dimensional space where eutonic calmness is construed as a state high in calmness (dimension 1) and high in energetic arousal (dimension 2).

Three more items were used to capture Qi Gong's specific impact as a form of meditative movement: Deep relaxation, meditative focus, and a heightened body awareness due to specific patterns of breathing and slow flowing movements. The items were construed by the first author based on the theoretical descriptions presented above [3]; (“I feel pleasure in my body,” “My attention is focused,” and “I can sense my own body”). All items in the respective languages are contained in the Additional file 2.

#### 2.2.2. Heart Rate Variability

The root mean square of successive beat-to-beat (RR) differences (RMSSD) and power of the high-frequency band (HF; 0.15–0.4 Hz) were taken as indicators of parasympathetic modulation [19]. Very-low frequency power (VLF; 0.0033–0.04 Hz) was tracked due to its known increase under physical activity and link to overall health [19, 44]. VLF and HF values were expected to significantly deviate from a normal distribution [20]; hence, the natural logarithm of the respective values was used (lnHF, lnVLF). Changes of RR-interval length over time were visually examined for rhythmic patterns of change in modulation. The standard deviation of the IBI normal-normal sinus beats (SDNN) was taken as the measure of overall modulation from both the sympathetic and the parasympathetic nervous system. Cardiac coherence (HR coherence) was calculated using the approach of McCraty and Childre [33]: (1) Identification of the maximum peak in the 0.04–0.26 Hz range of the power spectrum; (2) calculation of the integral of a window of 0.030 Hz centered on that peak; (3) calculation of the coherence ratio as: Peak Power/(Total Power–Peak Power).

#### 2.2.3. Covariates

Qi-Gong-specific beliefs, years of Qi Gong experience, and age were examined as potential covariates in this study. Due to a significant age difference between the two national groups, the influence of cultural background was assessed via the reexamination of the global effects in the two national subsamples. Qi-Gong-specific beliefs regarding the effect of Qi Gong, its relation to science, and the nature of Qi (vital energy) were assessed on two scales (“Belief in Qi” and “Belief in the scientific investigatablity of Qi”) using seven questions in a 7-point Likert-scale format (Don't agree at all–totally agree). The derivation of the scales and the respective items are contained in the Additional files 3 and 4. Results of the factor analysis are contained in the Additional files 5 and 6.

### 2.3. Design and Procedure

#### 2.3.1. Study Protocol

**Figure 1:**
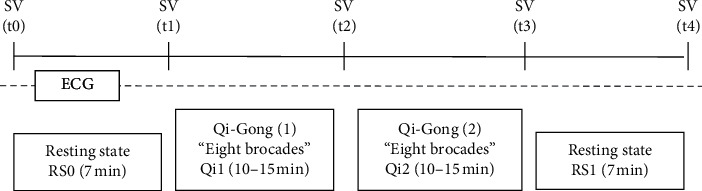
Study procedure and sequence of measurements. Measurement points to fill out self-report measures of subjective vitality (SV) and other subjective state variables before first rest (t0), after first rest (t1), after the first Qi Gong exercise (t2), after the second Qi Gong exercise (t3), and after second rest (t4). HRV parameters were calculated from the recorded electrocardiogram (ECG) for the periods of initial resting state in supine position (RS0), the first Qi Gong exercise (Qi1), the second Qi Gong exercise (Qi2), and the second resting state in supine position (RS1).

The study protocol consisted of four stages (Figure 1): an initial resting period in lying supine position (7 min), two subsequent performances of a standardized Qi Gong exercise (10 min) with a short rest in-between, and a post-exercise resting period in lying supine position (7 min). Prior to (t0, t1), in-between (t2), and after the Qi Gong exercise (t3, t4), the participants indicated their current subjective state on a self-report questionnaire (Additional file 2). Electrocardiogram (ECG) was recorded throughout the experiment using two nonreusable electrodes and a portable recording sensor (EcGMove 3, movisens GmbH) at a sampling rate of 1024 Hz. The measurement was conducted indoors and individually for each participant. Participants were given the choice to either come to a measurement room at the respective university or to perform the procedure at their usual training location (indoors). The standard moving Qi Gong exercise Baduanjin (“Eight sections of brocade”) consists of eight segments, which are repeated two to six times each; five of them bilaterally. The movements are performed in a slowly flowing manner and consist of twisting, stretching, and bending movements involving the whole body.

#### 2.3.2. Data Preprocessing

ECG data export from the sensor was done using Movisens SensorManager 1.8.130. The data were imported as ecg-file, visually checked for artifacts and analyzed using Kubios HRV Premium 3.0.2. For the two resting periods, the middle 5-min segments of the 7-min resting period were used. Due to technical issues and the Qi Gong typical stretching and twisting movements of the upper body, artifacts in at least one of the four segments were present in 17 participants. If possible, artifact-free segments ≥3 minutes were used as reliably representative of the respective segment [45]. In eight participants, we found that electrodes had been loosened or displaced during the practice. In two participants, the sensor recording only displayed noise. Overall, data from 10 participants (7 German, 3 Chinese) had to be excluded for the HRV analysis due to insufficient data quality Three participants had to be excluded from the analysis of the subjective measures due to missing answers. The dataset is available at: https://doi.org/10.17026/dans-x5h-ym26.

#### 2.3.3. Data Analysis

Repeated measures ANOVA were used to assess changes in subjective state and HRV indicators over the course of the measurement. Individual contrasts were examined using post-hoc tests. In order to examine the subjective state configuration induced by Qi Gong, we compared changes in all self-report items over the resting periods (t0-t1, t3-t4) with changes over the Qi Gong period (t0–t3, t1–t3). Correlational analysis was used to explore the relation between significant changes in subjective state variables (t0-t1, t1-t2, t1–t3, t3-t4) and HRV indicators (RS0, Qi1, Qi2, RS1, RS0-Qi1, RS0-Qi2).

To examine the influence of Qi Gong belief, Qi Gong experience in years and age, we examined the correlation between these variables and significant changes in subjective state and HRV parameters of the preceding analysis. Due to a significant difference in age between the two national subgroups, the influence of cultural background was examined via separate analysis for each national subgroup in addition to the combined analysis.

## 3. Results

### 3.1. Subjective State following Qi Gong

**Figure 2:**
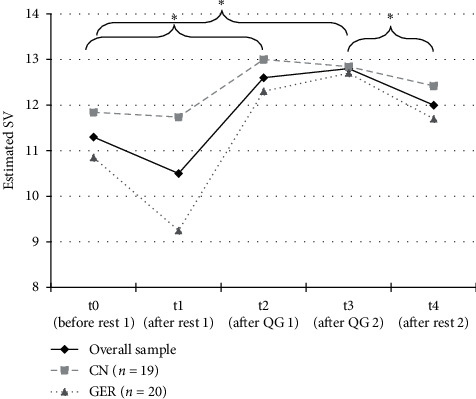
Changes in subjective vitality over rest and Qi Gong in the overall and the two national subsamples. Estimated means of self-reported subjective vitality (SV) before initial rest (t0, pre RS0), after initial rest (t1, post RS0), after the first Qi Gong exercise (t2, post QG1), after the second Qi Gong exercise (t3, QG2), and after second rest (t4, post RS1). Displayed are the means for the overall sample, the Chinese sample (CN), and the German sample (GER).

All subjective state variables displayed a significant change over the course of the experiment (*p* < 0.001). Overall, subjective vitality was significantly different between the different measurement points, *F*_(2.76, 105.02)_ = 19.79, *p* < 0.001, partial *η*^2^ = 0.34. Post-hoc tests revealed that SV significantly increased following the Qi Gong exercise compared to baseline (t0–t3; *p* < 0.001) and to the end of the resting period (t1–t3; *p* < 0.001). No significant change was observed during the initial resting period (t0-t1; *p* = .223). SV significantly decreased during the second resting period (t3-t4; *p* = 0.018) and returned to a level not significantly different from initial baseline (t0–t4; *p* = .64). The changes in SV over the course of the experiment in the overall sample and the national subsamples are displayed in Figure 2.

Repeated measures ANOVA showed that the subjective perception of calmness, *F*_(3.02,114.63)_ = 11.58, *p* < 0.001, partial *η*^2^ = 0.23; pleasant body sensations, *F*_(2.12, 80.61)_ = 11.66, *p* < 0.001, partial *η*^2^ = 0.23; focused attention, *F*_(2.84, 107.9)_ = 14.38, *p* < 0.001, partial *η*^2^ = 0.27; body awareness *F*_(2.77, 105.24)_ = 12.66, *p* < 0.001, partial *η*^2^ = 0.25; and perceived body activation, *F*_(2.63,99.96)_ = 24.58, *p* < 0.001, partial *η*^2^ = 0.39 significantly changed over the course of the experiment. The variables displayed different patterns of change: Calmness was the only measure that increased during the initial resting period (t0-t1; *p* = 0.002) and remained at this increased level (t0–t3; *p* = 0.001) over time until the end of the experiment (t1-t2, t2-t3; *p* > .10; t1–t4; *p* = .577). Pleasant body sensation, focused attention, body awareness, and perceived body activation did not change during either resting period (t0-t1, t3-t4; all *p* > .10) but increased over the course of the Qi Gong exercise compared to baseline (t0–t3; all *p* < 0.002) and remained at this level (t3-t4; all *p* > 0.05).

The sensation of Qi, *F*_(2.38,88.2)_ = 36.40, *p* < 0.001, partial *η*^2^ = 0.50 changed significantly over the course of the experiment. It did not change over the first resting period (t0-t1; *p* > .10) but increased following the Qi Gong exercises (t0–t3, t1–t3; all *p* < 0.001). The sensation of Qi significantly decreased over the second resting period (t3-t4; all *p* < 0.008). The estimated means, standard errors, and confidence intervals of all subjective state measures are displayed in Table 2. The detailed patterns of change are contained in Table 3. The general patterns of change for subjective state were similar in the Chinese (Table 4) and the German subsamples (Table 5) with stronger effects in the German sample. Further details on the subjective state analysis in the national subsamples can be found in Additional file 7 and Additional file 8.

### 3.2. Heart Rate Variability

All HRV parameters displayed a significant change over the course of the experiment (*p* < 0.05). The estimated means, standard errors, and confidence intervals of each parameter are reported in Table 6 (full descriptive HRV data are contained in the Additional file 9). Parameters of parasympathetic modulation showed a significant overall change during Qi Gong and rest, lnHF, *F*_(1.4,43.9)_ = 20.08, *p* < 0.001, partial *η*^2^ = 0.39; RMSSD, *F*_(1.2,38.2)_ = 14.93, *p* < 0.001, partial *η*^2^ = 0.32. Post-hoc tests revealed that both parameters significantly decreased during the first (RS0-QG1; *p* < 0.001) and the second Qi Gong exercises (RS0-QG2; *p* < 0.001) compared to the initial resting period. SDNN, *F*_(1.4,44.3)_ = 6.59, *p* = 0.006, partial *η*^2^ = 0.18; lnVLF, *F*_(1.8,55.0)_ = 31.71, *p* < 0.001, partial *η*^2^ = 0.51; and HR, *F*_(1.3,42)_ = 267.41, *p* < 0.001, partial *η*^2^ = 0.9 all displayed a significant overall effect. Post-hoc tests revealed a significant increase in all three parameters during Qi Gong compared to the resting conditions (all *p* < 0.006). No significant changes between the resting conditions (RS0-RS1) and between the Qi Gong conditions (Qi1-Qi2) were found.

**Figure 3:**
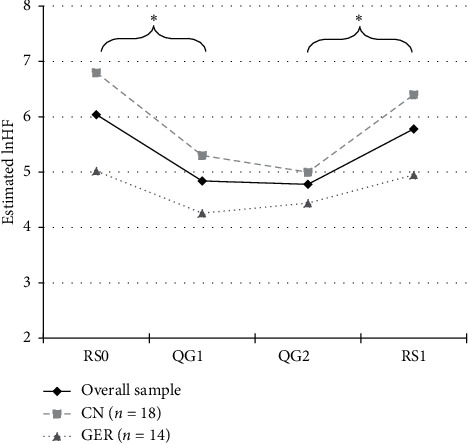
Changes in parasympathetic modulation over rest and Qi Gong in the overall and the two national subsamples. Estimated means of parasympathetic modulation (lnHF; natural logarithm of power in the high-frequency band) during initial rest (RS0), first Qi Gong (QG1), 2nd Qi Gong (QG2), and 2nd rest (RS1). Displayed are the means for the overall sample, the Chinese sample (CN), and the German sample (GER).

Changes of HRV indicators followed a similar descriptive trend in the Chinese (Table 7) and German subsamples (Table 8); however, overall effects for lnHF (*p* = .103), RMSSD (*p* = .106), and SDNN (*p* = .156) did not reach significance in the German sample.  Figure 3 displays exemplary changes in lnHF over rest and Qi Gong in the overall and the national subsamples. Further details on the analysis can be found in the Additional file 10.

### 3.3. Coherence

**Figure 4:**
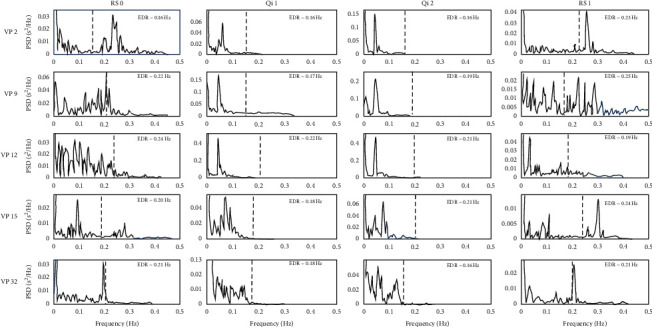
Frequency power distribution in five sample participants. Power spectra of five different participants during initial rest (RS 0), first Qi Gong (Qi 1), second Qi Gong (Qi (2), and second rest (RS 1). VP 2, VP 9, and VP 12 from the Chinese sample are examples that display a high degree of VLF band corrected (<0.004 Hz) coherence (SP coherence) at a peak around 0.05 Hz. VP 15, also from the Chinese sample, is an example for medium SP coherence and VP 32 from the German sample displays no SP coherence.

No significant overall change in HR coherence was found (*p* = .14). Visual inspection of the power distribution graphs showed a peak around the frequency of 0.6 Hz in the LF band and a peak in the VLF band of multiple participants (Figure 4). Using a spline interpolation (SP) filter for all frequencies <0.04 Hz (VLF), a significant overall difference in spline-interpolated cardiac coherence (SP coherence) resulted, *F*_(1.6,49.7)_ = 4.35, *p* = 0.025, partial *η*^2^ = 0.12. Post-hoc contrasts revealed SP coherence to be significantly higher during the second Qi Gong exercise compared to the resting baseline (RS0-Qi2; *p* = 0.011). SP coherence during the second rest did not deviate significantly from SP coherence during initial rest (RS0-RS1; *p* = 0.131).

The effect for SP coherence was only found in the Chinese sample *F*_(1.3, 22.3)_ = 6.37, *p* = 0.013, partial *η*^2^ = 0.27, whereas the German sample did not display an overall difference between conditions (*p* = 0.57).

### 3.4. Subjective State and HRV Explorative Correlation

#### 3.4.1. Overall Sample

No significant correlations were found between changes in subjective state variables (t1-t2, t1-t3) and changes in HRV parameters (RS0-Qi1, RS0-Qi2) or HRV parameters (Qi1, Qi2) in the overall sample. Decreases in perceived body activation (t3-t4) during second rest (RS1) were stronger in participants with higher RMSSD, *r* = 0.33, *p* = 0.046, higher lnHF, *r* = 0.33, *p* = 0.048, and lower heart rate, *r* = −0.43, *p* = 0.003.

#### 3.4.2. National Subsamples

In the German sample, a stronger increase in heart rate during the first Qi Gong (RS-Qi1) was associated with a stronger increase (t1-t2) in subjective vitality, *r* = 0.52, *p* = 0.045, perceived body activation, *r* = 0.67, *p* = 0.006 and perception of Qi, *r* = 0.82, *p* < 0.001. A stronger increase in heart rate during the second Qi Gong (RS0-Qi2) correlated with a stronger increase in body awareness (t1–t3), *r* = 0.57, *p* = 0.02 (RS0-Qi2). Decreases in perceived body activation (t3-t4) during the second rest were stronger in participants with higher RMSSD, *r* = 0.65, *p* = 0.006, higher lnHF, *r* = 0.52, *p* = 0.04 and lower HR, *r* = −0.67, *p* = 0.004. Furthermore, decreases in subjective vitality over the second rest (t3-t4) were stronger in participants with higher RMSSD, *r* = 0.59, *p* = 0.015.

In the Chinese subsample, participants who showed a higher increase in subjective vitality (t1-t2) after the first Qi Gong exercise also showed a stronger decrease in RMSSD, *r* = −0.42, *p* = 0.064. A stronger increase in body awareness (t1–t3) was correlated with decrease of lnHF, *r* = −0.51, *p* = 0.03 (RS0-Qi2). High SP coherence during the second Qi Gong exercise (Qi2) correlated with increases (t1–t3) in subjective vitality, *r* = −0.46, *p* = 0.056 and perception of Qi, *r* = −0.51, *p* = 0.03.

### 3.5. Covariates: Beliefs Experience, Age

Participants who believed more in Qi showed a higher increase in attentional focus, *r* = −0.33, *p* = 0.037 (t1-t2), *r* = −0.37, *p* = 0.014 (t1–t3) and stronger decrease in body activation during the second rest, *r* = −0.32, *p* = 0.038 (t3-t4). Belief in the accessibility of Qi to scientific investigation correlated with less perception of Qi, *r* = 0.34, *p* = 0.028 (t1-t3) following Qi Gong. Years of Qi Gong experience did not correlate significantly with any changes in the subjective state in the overall sample. Older participants showed a significantly higher increase in SV following Qi Gong (t1–t3), *r* = −0.44, *p* = 0.003.

Participants with a stronger belief in Qi showed a significantly higher increase in SDNN, *r* = −0.33, *p* = 0.047 (RS0-Qi1). Subjects with a stronger belief in the accessibility of Qi to scientific investigation showed less increase in lnVLF, *r* = 0.36, *p* = 0.037 (RS0-Qi1) during the first Qi Gong exercise. Older participants showed significantly less decrease in measures of vagal modulation (RMSSD, lnHF): RMSSD, *r* = −0.51, *p* = 0.001 (RS0-Qi1), *r* = −0.59, *p* < 0.001 (RS0-Qi2); lnHF, *r* = −42, *p* = 0.011 (RS0-Qi1), *r* = −.54, *p* < 0.001 (RS0-Qi2) and less increase in HR, *r* = 0.68, *p* < 0.001 (RS0-Qi1), *r* = 0.70, *p* ≤ 0.001 (RS0-Qi2). Qi Gong experience in years showed weaker correlations for the same variables as age; however, when controlled for age, none of the correlations remained significant (all *p* > .10).

## 4. Discussion

The overall goal of this study was to explore the psychophysiological mechanism underlying mind-body exercise using the example of a standard moving Qi Gong exercise (Baduanjin). Two bouts of Baduanjin effectively induced a state of eutonic calmness, characterized by high calmness and perceived body activation, increased subjective vitality, attentional focus and body awareness, accompanied by pleasurable body sensations. On a physiological level, parasympathetic modulation (lnHF, RMSSD) significantly decreased, whereas overall modulation (SDNN), physiological activation (lnVLF and heart rate), and SP coherence significantly increased during Qi Gong.

### 4.1. A Mechanism of General Activation

Our findings of a decrease in parasympathetic modulation during the exercise are in line with views of Qi Gong as moderate aerobic exercise [10, 11] and its acute effects [46–48] potentially needed for long-term health adaption processes [49, 50]. The findings support Qi Gong's assumed capacity for moderate upregulation (activation of the ANS) as one component to reach a state of eutonic calmness [4]. Activation of the ANS is used as a general term for physiological activation here because HRV parameters do not allow for a distinction between activation due to parasympathetic withdrawal and activation due to increased sympathetic nervous system activity. From a general health perspective, the mechanism of moderate activation highlights a route to explain long-term beneficial effects of mind-body exercise on hypo-aroused conditions such as fatigue [12] and depression [13]. One interesting point is that the average HR in this study did not raise above levels of moderate activation (>100BPM), which can be achieved solely by parasympathetic withdrawal [51, 52], Activation due to parasympathetic withdrawal during Qi Gong may comprise benefits of moderate activation without potential draining effects of top-down modulated sympathetic activation [23, 53, 54] and be a characteristic of the health beneficiary effects of Qi Gong.

On the experiential level, changes in parasympathetic modulation (RMSSD, lnHF) displayed negative, indicators of activation (HR, lnVLF), positive correlations with perceived body activation, and subjective vitality. These findings underline the general activating aspect of body-mind exercise via its influence on energy [55] and subjective vitality as activated positive emotion [56–58].

### 4.2. A Mechanism of Microregulation

**Figure 5:**
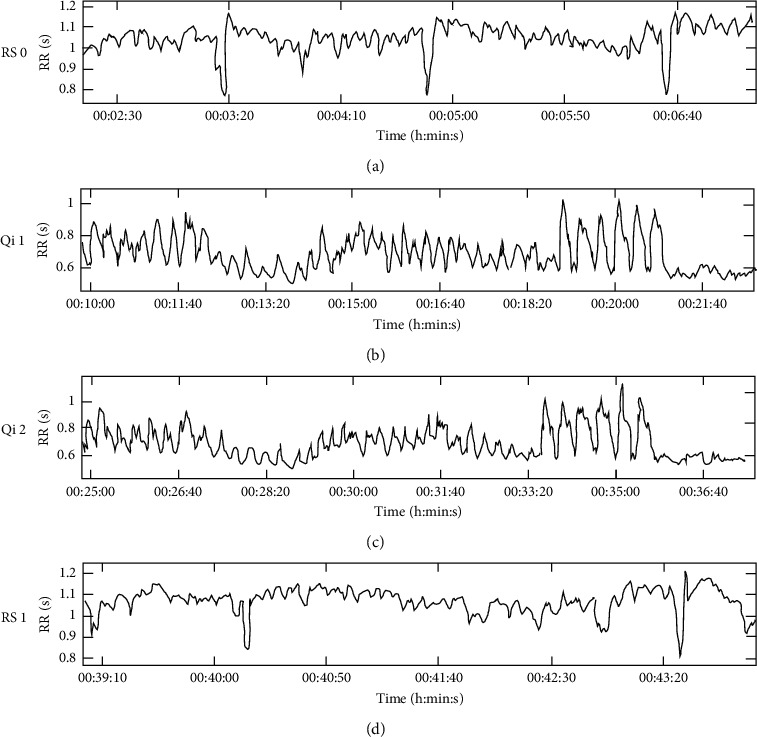
Sample RR-interval trajectory during rest and Qi Gong. RR-interval series of VP12 for initial rest (RS 0), the first Qi Gong exercise (Qi 1), the second Qi Gong exercise (Qi 2) and the resting period following Qi Gong (RS 1). VP12 displayed a high degree of SP coherence (Figure 4). This figure shows the rhythmic changes (around 0.05 Hz) in RR intervals associated with activation and relaxation during both Qi Gong exercises.

Regulation, as in the context of this study, emphasizes the conjunct presence of ANS activation (upregulation) and relaxation (downregulation). The rhythmic rise and decline in RR-interval length, displayed for an exemplary subject in Figure 5, indicates such a pattern of microregulation. The pattern drives the significant increase in the overall standard deviation of RR intervals (SDNN) and its regularity displays in the significant increase of SP coherence at a peak frequency between 0.05 Hz and 0.07 Hz. This frequency aligns with rhythmic changes in movement and potentially breath and inner imagery [7] during Qi Gong in this study: The eight segments of Baduanjin Qi Gong each consist of an “upregulation” (e.g., lifting arms palms facing the sky, breathing in) and a “downregulation” (e.g., falling arms at the side of the body, breathing out) part, repeated six times per segment. In the example of the Chinese subgroup, this leads to a calculated frequency of 0.06 Hz (12:19 min/48 movements) for the second Qi Gong exercise that matches SP coherence peak between 0.05 Hz and 0.07 Hz. Increases in VLFband activity initially covered a significant increase in coherence; however, they are in line with effects of exercise on the power spectrum [44].

Based on the preceding analysis, in this study, the increase in SDNN can be interpreted as a marker of overall increase in regulation. As other forms of exercise tend to show a decrease in SDNN [48], the increase in SDNN displays a potential characteristic of body-mind exercise. Studies that find stable increases in SDNN following long-term Qi Gong interventions provide initial evidence for the transference and stability of this increased regulative capacity [24, 28]. Overall, we suggest that regulation via bottom-up ANS pacemaking in Qi Gong invites healthy oscillating capacity in the organism [53, 59] and comprises a characteristic mechanism of body-mind exercise.

On the subjective level, regulation links to the dimension of calmness [55] and subjective vitality as marker of regulative capacity [36]. Changes in these variables, as well as the increase in attentional focus, the awareness of one's own body and pleasurable body feelings [3] confirm the capacity of mind-body exercises to reach a state of eutonic calmness or relaxed attentiveness with a body focus [4, 56].

### 4.3. Multicomponent Intervention: Covariates and National Subsamples

Overall, the decrease of parasympathetic modulation (lnHF, RMSSD) and increase of lnVLF band power was less pronounced in older participants. This is in line with age-related changes of HRV during rest and exercise in general [20, 46]. Furthermore, the finding offers an explanation for inconclusive results regarding resting state HRV changes following long-term Baduajin training protocols in different age groups [25, 27, 29].

Regarding the difference between the two samples, the significant higher age in the German subsample in this study (56.3 vs 28.8) may have been a reason for the finding of less pronounced changes in HRV parameters and differences in subjective state physiology correlations. Furthermore, the majority of the German participants (*n* = 17) were recreational practitioners compared to the Chinese sample with a professional Qi Gong training background (University degree). The difference in SP coherence increase could have been the result of differing amounts of deliberate practice [60]. In line with this argument, in the Chinese subsample, years of Qi Gong experience correlated significantly with increase in body awareness (t1−t3: r = −0.70, *p* < 0.001), perception of Qi (t1-t2: r = −0.47, *p* = 0.031), and subjective vitality (t1−t3: r = −0.44, *p* = 0.045) beyond the explanatory power of age, which matches findings of body awareness as marker of expertise in body-mind exercise [61]. The significant influence of Qi-Gong-specific beliefs found in this study confirmed Qi Gong's nature as a multicomponent intervention [40, 41].

### 4.4. Limitations

This study had a number of limitations: The use of a quasi-experimental design in this study limits the possibilities for causal conclusions. Due to the significant difference in age, no direct comparison between the two subsamples was possible. Furthermore, participants were allowed to take the measurements in their usual training location or at the respective university. In this way, potential effects of environmental factors could not be ruled out. A further limitation was the limited recording time following the completion of the second resting period. We found that most parameters of subjective state and all HRV parameters returned to baseline during the second resting period, hence a latent change in one of the parameters could not have been detected in this design. Furthermore, to specifically measure the regulative effect of Qi Gong, future studies should use passive (i.e., standing) and active (i.e., walking) as well as hypo-aroused (i.e., autogenous training) and hyper-aroused (i.e., stress task) control conditions.

## 5. Conclusion

Overall, we found that the Qi Gong form of Baduanjin reliably induced a state of eutonic calmness associated with moderate physiological activation and rhythmic microregulation within the ANS. The subjective state following Qi Gong entails a specific profile of calmness and perceived body activation linked to attentional focus, body awareness, pleasant body sensation, and subjective vitality. Linking back to the initial question, the findings suggest a mechanism of moderate general ANS activation in combination with an exercise-dependent pattern of microregulation via movement, breath, and imagery to be the health beneficial characteristic of body-mind exercise.

## Figures and Tables

**Table 1:** Demographic variables and descriptive statistics for the overall samples and subsamples.

	Overall sample *N* = 42		Chinese sample *n* = 21		German sample *n* = 21		Test statistics (comparison of national subsamples)
	Mean	SD	Mean	SD	Mean	SD	t/*χ*^2^
Age	42.60	18.30	28.81	11.56	56.38	12.4	t_40_ = 7.45 *p* < 0.001

Sex (f/m)	67.4% 32.6%		61.9% 38.1%		76.2% 23.8%		*χ*_1_^2^ = 1.00 *p* > 0.1

Education							*χ*_2_^2^ = 12.35 *p* = 0.002
(i) Middle school	18.6%				38.1%		
(ii) High school	18.7%		14.3%		23.8%		
(iii) University	62.8%		85.7%		38.1%		

Qi Gong experience							
Years	8.54	5.97	5.64	4.38	11.43	6.02	t_36.53_ = 3.56 *p* = 0.001
∅ days p. week	3.34	1.62	3.81	1.4	2.88	1.72	t_40_ = 1.92 *p* = 0.063
∅ minutes p. practice	53.74	50.48	77.24	58.25	30.24	25.91	t_27.61_ = 3.38 *p* = 0.002

Duration of Qigong exercise in the experiment							
Qi Gong 1 (min)	10:38	02:37	12:18	01:45	08:58	02:21	t_40_ = 5.18 *p* < 0.001
Qi Gong 2 (min)	10:47	02:22	12:19	01:20	09:16	02:15	t_40_ = 5.34 *p* < 0.001

Average days of Qi Gong practice per week (∅ days *p*. week); average duration in minutes per practice (∅ minutes *p*. practice).

**Table 2:** Estimated means, standard errors, confidence intervals, and test statistics for all subjective state measures in the overall sample.

Overall sample (*N* = 39)	t0	SE CI	t1	SE CI	t2	SE CI	t3	SE CI	t4	SE CI	*F*	*p*
Subjective vitality	11.3	0.34[10.6; 12.0]	10.5	0.39 [9.7; 11.2]	12.6	0.27 [12.1; 13.2]	12.8	0.27 [12.2; 13.3]	12.0	0.33 [11.4; 12.8]	19.79	<0.001
Calmness	5.6	0.18 [5.2; 6.0]	6.2	0.14 [6.0; 6.5]	6.1	0.14 [5.8; 6.3]	6.4	0.15 [6.1; 6.7]	6.5	0.13 [6.2; 6.8]	11.58	<0.001
Pleasant body sensation	5.5	0.23 [5.0; 5.9]	5.9	0.21 [5.4; 6.3]	6.3	0.13 [6.0; 6.5]	6.4	0.13 [6.2; 6.7]	6.5	0.13 [6.3; 6.8]	11.66	<0.001
Focused attention	5.6	0.2 [5.2; 6.0]	5.8	0.18 [5.5; 6.2]	6.3	0.12 [6.1; 6.6]	6.4	0.14 [6.2; 6.7]	6.4	0.12 [6.2; 6.6]	14.38	<0.001
Body awareness	5.9	0.16 [5.5; 6.2]	6.0	0.17 [5.6; 6.3]	6.5	0.1 [6.3; 6.7]	6.6	0.12 [6.4; 6.8]	6.5	0.11 [6.3; 6.7]	12.66	<0.001
Perceived body activation	5.1	0.22 [4.7; 5.6]	4.9	0.25 [4.4; 5.4]	6.4	0.11 [6.1; 6.6]	6.5	0.12 [6.3; 6.8]	6.0	0.21 [5.6; 6.4]	24.58	<0.001
Perception of Qi	4.3	0.27 [3.7; 4.8]	4.8	0.27 [4.3; 5.4]	6.0	0.17 [5.7; 6.4]	6.3	0.18 [5.9; 6.7]	5.9	0.18 [5.5; 6.2]	36.4	<0.001

Estimated means, standard errors (SE), and 95% confidence intervals (CI) are reported for all subjective state variables at times t0 (before initial rest), t1 (after initial rest), t2 (after first Qi Gong), t3 (after 2nd Qi Gong), and t4 (after 2nd rest).

**Table 3:** Changes in subjective vitality and subjective state measures during rest and Qi Gong in the overall sample.

Overall sample (*N* = 39)	Rest 0 (t0-t1)	Qi Gong (t0–t3)	Qi Gong t1-t3	Rest 1 (t3-t4)	Overall *F*
Subjective vitality	**O** 0.223	**↗** <0.001	**↗** <0.001	**↘** 0.018	19.79 <0.001

Calmness	**↗** 0.002	**↗** 0.001	**O** >0.99	**O** >0.99	11.58 <0.001

Pleasant body sensation	**O** 0.163	**↗** 0.002	**O** 0.052	**O** >)0.99	11.66 <0.001

Focused attention	**O** 0.864	**↗** <0.001	**↗** 0.004	**O** >0.99	14.38 <0.001

Body awareness	**O** >0.99	**↗** <0.001	**↗** <0.001	**O** >0.99	12.66 <0.001

Perceived body activation	**O** >0.99	**↗** <0.001	**↗** <0.001	**O** 0.051	24.58 <0.001

Sensation of Qi	**O** 0.118	**↗** <0.001	**↗** <0.001	**↘** 0.003	36.4 <0.001

Results for post-hoc comparisons are reported for the change over the first resting period (Rest 0, t0-t1), the difference between initial baseline and following 2nd Qi Gong (Qi Gong, t0–t3), the difference after first rest and after 2nd Qi Gong (Qi Gong, t1–t3), and the difference before and after the 2nd resting period (Rest 1, t3-t4). ↗ significant increase, ↘ significant decrease at *α* = 0.05; O no significant change, *p*-values of post-hoc test reported underneath.

**Table 4:** Estimated means, standard errors, confidence intervals, and test statistics for all subjective state measures in the Chinese sample.

Chinese sample (*n* = 19)	t0	SE CI	t1	SE CI	t2	SE CI	t3	SE CI	t4	SE CI	*F*	*P*
Subjective vitality	11.8	0.4 [11.0; 12.7]	11.7	0.34 [11.0; 12.4]	13.0	0.35 [12.3; 13.8]	12.8	0.31 [12.2; 13.5]	12.4	0.35 [11.7; 13.2]	4.92	=0.01^*∗*^
Calmness	5.9	0.19 [5.5; 6.3]	6.5	0.16 [6.2; 6.9]	6.2	0.23 [5.7; 6.5]	6.5	0.19 [6.1; 6.9]	6.5	0.23 [6.0; 7.0]	3.91	=0.019^*∗*^
Pleasant body sensation	5.7	0.36 [5.0; 6.5]	6.3	0.22 [5.9; 6.8]	6.3	0.2 [5.9; 6.7]	6.5	0.18 [6.1; 6.8]	6.5	0.19 [6.1; 6.9]	2.54	=0.101
Focused attention	5.8	0.26 [5.2; 6.3]	6.2	0.2 [5.8; 6.6]	6.5	0.19 [6.1; 6.9]	6.6	0.17 [6.3; 7.0]	6.4	0.16 [6.0; 6.7]	5.76	=0.006^*∗*^
Body awareness	6.1	0.21 [5.7; 6.6]	6.4	0.16 [6.0; 6.7]	6.7	0.13 [6.4; 7.0]	6.8	0.09 [6.7; 7.0]	6.5	0.18 [6.1; 6.8]	4.95	=0.009^*∗*^
Perceived body activation	5.5	0.3 [4.8; 6.1]	5.7	0.26 [5.1; 6.2]	6.5	0.14 [6.2; 6.8]	6.7	0.13 [6.4; 7.0]	6.2	0.22 [5.7; 6.7]	8.56	<0.001^*∗*^
Perception of Qi	5.1	0.29 [4.5; 5.7]	5.5	0.27 [5.0; 6.1]	6.2	0.27 [5.6; 6.8]	6.4	0.26 [5.8; 6.9]	5.8	0.24 [5.3; 6.3]	14.01	<0.001^*∗*^

Estimated means, standard errors (SE), and 95% confidence intervals (CI) are reported for all subjective state variables at times t0 (before initial rest), t1 (after initial rest), t2 (after first Qi Gong), t3 (after 2nd Qi Gong), and t4 (after 2nd rest).

**Table 5:** Estimated means, standard errors, confidence intervals, and test statistics for all subjective state measures in the German sample.

German sample (*n* = 20)	t0	SE CI	t1	SE CI	t2	SE CI	t3	SE CI	t4	SE CI	*F*	*p*
Subjective vitality	10.8	0.54 [9.7; 12.0]	9.2	0.58 [8.0; 10.5]	12.3	0.4 [11.5; 13.0]	12.7	0.45 [11.7; 13.6]	11.7	0.56 [10.5; 12.9]	18.21	<0.001^*∗*^
Calmness	5.3	0.3 [4.7; 6.0]	6.0	0.2 [5.6; 6.4]	6.0	0.16 [5.7; 6.3]	6.2	0.22 [5.7; 6.7]	6.5	0.13 [6.3; 6.8]	9.43	<0.001^*∗*^
Pleasant body sensation	5.2	0.3 [4.6; 5.8]	5.4	0.33 [4.8; 6.1]	6.2	0.18 [5.9; 6.6]	6.4	0.18 [6.0; 6.8]	6.5	0.17 [6.1; 6.8]	13.41	<0.001^*∗*^
Focused attention	5.4	0.29 [4.8; 6.0]	5.5	0.28 [4.9; 6.1]	6.2	0.16 [5.9; 6.5]	6.2	0.2 [5.8; 6.7]	6.4	0.15 [6.1; 6.7]	10.34	<0.001^*∗*^
Body awareness	5.6	0.24 [5.1; 6.1]	5.6	0.27 [5.0; 6.2]	6.3	0.15 [6.0; 6.7]	6.4	0.21 [6.0; 6.8]	6.5	0.15 [6.2; 6.9]	10	<0.001^*∗*^
Perceived body activation	4.8	0.31 [4.1; 5.4]	4.2	0.35 [3.5; 4.9]	6.2	0.17 [5.8; 6.6]	6.3	0.2 [5.9; 6.8]	5.8	0.34 [5.1; 6.6]	18.4	<0.001^*∗*^
Perception of Qi	3.5	0.36 [2.7; 4.2]	4.1	0.42 [3.2; 5.0]	5.9	0.2 [5.5; 6.3]	6.2	0.27 [5.6; 6.8]	5.9	0.27 [5.3; 6.5]	31.22	<0.001^*∗*^

Estimated means, standard errors (SE), and 95% confidence intervals (CI) are reported for all subjective state variables at times t0 (before initial rest), t1 (after initial rest), t2 (after first Qi Gong), t3 (after 2nd Qi Gong), and t4 (after 2nd rest).

**Table 6:** Estimated means, standard errors, and 95% confidence intervals for all HRV parameters.

Overall sample (*N* = 32)	RS_0	SE CI	Qi_1	SE CI	Qi_2	SE CI	RS_1	SE CI	*F*	*p*
lnHF	6.04	0.29 [5.45; 6.62]	4.84	0.22 [4.39; 5.29]	4.78	0.2 [4.37; 5.2]	5.78	0.28 [5.21; 6.36]	20.01	<0.001^*∗*^
lnVLF	6.17	0.17 [5.83; 6.51]	7.37	0.18 [7.0; 7.74]	7.45	0.18 [7.1; 7.81]	6.3	0.19 [5.93; 6.7]	31.71	<0.001^*∗*^
Coherence	0.43	0.06 [0.3; 0.56]	0.49	0.06 [0.36; 0.61]	0.49	0.07 [0.36; 0.63]	0.34	0.05 [0.24; 0.43]	1.97	=0.14
SP coherence	0.79	0.12 [0.55; 1.04]	1.38	0.33 [0.71; 2.05]	1.35	0.13 [1.08; 1.63]	0.64	0.09 [0.46; 0.82]	4.35	=0.025^*∗*^
RMSSD	49.43	6.71 [35.74; 63.12]	22.96	1.81 [19.26; 26.66]	23.69	1.18 [19.91; 27.48]	47.77	7.67 [32.12; 63.42]	14.93	<0.001^*∗*^
SDNN	52.74	5.09 [42.36; 63.11]	65.26	4.53 [56.01; 74.51]	65.81	4.27 [57.11; 74.51]	53.04	5.61 [41.6; 64.48]	6.86	=0.006^*∗*^
HR	66.46	1.67 [63; 69.93]	89.85	1.49 [86.81; 92.9]	91.78	1.63 [88.45; 95.1]	65.95	1.6 [62.69; 69.22]	267.41	<0.001^*∗*^

The variables are the natural logarithm of high-frequency band (lnHF), the natural logarithm of the very low frequency band (lnVLF), coherence, the spline-interpolated coherence (SP coherence; all frequencies <0.004 Hz excluded), the root mean square of successive differences between heart beats (RMSSD), the standard deviation of the normal (NN) inter-beat intervals (SDNN), and heart rate (HR).

**Table 7:** Estimated means, standard errors, and 95% confidence intervals for all HRV parameters in the Chinese sample.

Chinese sample (*n* = 18)	RS0	SE CI	Qi1	SE CI	Qi2	SE CI	RS1	SE CI	*F*	*p*
lnHF	6.8	0.32 [6.16; 7.5]	5.3	0.2 [4.88; 5.71]	5.0	0.2 [4.63; 5.48]	6.4	0.33 [5.75; 7.12]	25.75	<0.001^*∗*^
lnVLF	6.5	0.18 [6.12; 6.88]	8.04	0.13 [7.75; 8.83]	8.07	0.13 [7.8; 8.35]	6.6	0.25 [6.07; 7.13]	36.57	<0.001^*∗*^
Coherence	0.39	0.08 [0.21; 0.57]	0.46	0.06 [0.33; 0.6]	0.42	0.07 [0.28; 0.57]	0.24	0.04 [0.15; 0.33]	2.24	=0.118
SP coherence	0.61	0.16 [0.27; 0.95]	1.75	0.48 [0.74; 2.75]	1.38	0.12 [1.13; 1.64]	0.4	0.07 [0.26; 0.54]	6.37	=0.013^*∗*^
RMSSD	63.99	9.43 [44.1; 83.89]	26.28	1.96 [22.16; 30.41]	24.53	1.51 [21.34; 27.73]	62.43	11.08 [39.04; 85.81]	14.81	=0.001^*∗*^
SDNN	63.45	6.75 [49.21; 77.68]	80.79	4.44 [71.43; 90.15]	78.57	4.12 [69.88; 87.25]	60.86	7.97 [44.05; 77.67]	5.67	=0.018^*∗*^
HR	62.08	1.64 [58.63; 65.54]	89.48	2.06 [85.13; 93.82]	92.69	2.19 [88.08; 97.31]	63.22	1.64 [59.77; 66.67]	214.8	<0.001^*∗*^

The variables are the natural logarithm of high-frequency band (lnHF), the natural logarithm of the very low frequency band (lnVLF), coherence, the spline-interpolated coherence (SP coherence; all frequencies <0.004 Hz excluded), the root mean square of successive differences between heart beats (RMSSD), the standard deviation of the normal (NN) inter-beat intervals (SDNN), and heart rate (HR).

**Table 8:** Estimated means, standard errors, and 95% confidence intervals for all HRV parameters in the German sample.

German sample (*n* = 14)	RS0	SE CI	Qi1	SE CI	Qi2	SE CI	RS1	SE CI	*F*	*p*
lnHF	5.02	0.38 [4.2; 5.83]	4.26	0.38 [3.41; 5.11]	4.44	0.38 [3.61; 5.26]	4.95	0.4 [4.08; 5.82]	2.7	=0.103
lnVLF	5.75	0.27 [5.16; 6.33]	6.52	0.23 [6.02; 7.01]	6.65	0.24[6.13; 7.16]	5.9	0.26 [5.37; 6.52]	5.34	=0.012^*∗*^
Coherence	0.48	0.1 [0.26; 0.7]	0.51	0.11 [0.28; 0.75]	0.59	0.12 [0.32; 0.85]	0.46	0.09 [0.27; 0.65]	0.42	=0.669
SP coherence	1.03	0.16 [0.69; 1.37]	0.9	0.42 [0; 1.8]	1.3	0.27 [0.73; 1.9]	0.96	0.15 [0.64; 1.28]	0.6	=0.57
RMSSD	30.71	6.94 [15.72; 45.71]	18.69	3 [12.21; 25.17]	22.61	3.84 [14.32; 30.9]	28.92	8.1 [11.41; 46.42]	2.66	=0.106
SDNN	38.96	6.2 [25.56; 52.37]	45.29	4.94 [34.62; 55.97]	49.41	5.83 [36.82; 62.01]	42.98	7.13 [27.56; 58.39]	2.09	=0.156
HR	72.1	2.62 [66.43; 77.76]	90.34	2.24 [85.5; 95.18]	90.6	2.5 [85.2; 96.0]	69.47	2.79 [63.43; 75.5]	177.56	<0.001^*∗*^

The variables are the natural logarithm of high-frequency band (lnHF), the natural logarithm of the very low frequency band (lnVLF), coherence, the spline-interpolated coherence (SP coherence; all frequencies <0.004 Hz excluded), the root mean square of successive differences between heart beats (RMSSD), the standard deviation of the normal (NN) inter-beat intervals (SDNN), and heart rate (HR).

## Data Availability

The datasets generated and analyzed during the current study are available in the DANS repository (https://doi.org/10.17026/dans-x5h-ym26 [62]).

## Abbreviations

TCM: Traditional Chinese medicine
ANS: Autonomous nervous system
HRV: Heart rate variability
LF: Low frequency
HF: High frequency
VLF: Very low frequency
RMSSD: Root mean square of successive R-R differences
LnHF: Logarithm of the high frequency
LnLF: Logarithm of the low frequency
SDNN: Standard deviation of normal-to-normal beat intervals
HR coherence: Cardiac coherence
SP coherence: Spline-interpolated cardiac coherence.

## Supplementary Materials

Supplementary Materials Additional files. Additional file 1 (docx): National subsample characteristics. Additional file 2 (docx): Subjective state items in English, Chinese, and German. Additional file 3 (docx): Generation and factor-scale analysis of Qi belief items. Additional file 4 (docx): Belief items in English, Chinese, and German. Additional file 5 (docx): Rotated factor loadings, Eigenvalue, and Cronbach's Alpha of all belief items. Additional file 6 (docx): Rotated factor loadings, Eigenvalue, and Cronbach's Alpha of selected belief items. Additional file 7 (docx): Changes in subjective state over experiment in overall and national subsamples. Additional file 8 (docx): Subjective state changes (national subsamples). Additional file 9 (docx): Heart rate variability descriptive data (overall sample). Additional file 10 (docx): HRV analysis (national subsamples).
