# Supplementary material for: Relaxation or Regulation: The Acute Effect of Mind-Body Exercise on Heart Rate Variability and Subjective State in Experienced Qi Gong Practitioners
Source: Evid Based Complement Alternat Med. 2021 Jun 8;2021:6673190. doi: 10.1155/2021/6673190 (PMC8208883; doi:10.1155/2021/6673190)
Supplement: Supplementary Materials — Additional files. Additional file 1 (docx): National subsample characteristics. Additional file 2 (docx): Subjective state items in English, Chinese, and German. Additional file 3 (docx): Generation and factor-scale analysis of Qi belief items. Additional file 4 (docx): Belief items in English, Chinese, and German. Additional file 5 (docx): Rotated factor loadings, Eigenvalue, and Cronbach's Alpha of all belief items. Additional file 6 (docx): Rotated factor loadings, Eigenvalue, and Cronbach's Alpha of selected belief items. Additional file 7 (docx): Changes in subjective state over experiment in overall and national subsamples. Additional file 8 (docx): Subjective state changes (national subsamples). Additional file 9 (docx): Heart rate variability descriptive data (overall sample). Additional file 10 (docx): HRV analysis (national subsamples). [file 6673190.f1.zip › 6673190.f1/Additional file 1.docx]

**National subsample characteristics**

The Chinese and the German sample deviated significantly from each other with regard to age, t_40_=7.45 p < .001, and level of education, χ^2^_2_=12.35 p=.002. The mean age in the German sample was 56.4 years (*SD*=12.4) and 28.8 years (*SD*=11.6) in the Chinese sample. Chinese participants indicated marginally significant more occasions of practice per week, t_40_=1.92, p =.063 and on average a longer duration of practice t_27.61_=3.38, p =.002 whereas German participants reported a significantly longer experience in average years of Qi Gong practice, t_36.53_=3.56, p =.001. As a result, the sub-samples did not differ in the estimated overall time spent on Qi Gong practice (p > .10). The subsamples showed no difference in the belief in the existence and power of Qi. A significant difference between groups was found for the belief in the accessability of Qi to scientific investigation, t(40) = 2.628, p = .012 with the Chinese sample believing more strongly in this aspect. Chinese participants differed significantly from German participants in the duration of both exercise rounds, t_40_=5.183, p <.001/t_40_=5.341, p <.001 with the Chinese sample taking an average of 3:19min/3:03min longer.
