# Supplementary material for: Relaxation or Regulation: The Acute Effect of Mind-Body Exercise on Heart Rate Variability and Subjective State in Experienced Qi Gong Practitioners
Source: Evid Based Complement Alternat Med. 2021 Jun 8;2021:6673190. doi: 10.1155/2021/6673190 (PMC8208883; doi:10.1155/2021/6673190)
Supplement: Supplementary Materials — Additional files. Additional file 1 (docx): National subsample characteristics. Additional file 2 (docx): Subjective state items in English, Chinese, and German. Additional file 3 (docx): Generation and factor-scale analysis of Qi belief items. Additional file 4 (docx): Belief items in English, Chinese, and German. Additional file 5 (docx): Rotated factor loadings, Eigenvalue, and Cronbach's Alpha of all belief items. Additional file 6 (docx): Rotated factor loadings, Eigenvalue, and Cronbach's Alpha of selected belief items. Additional file 7 (docx): Changes in subjective state over experiment in overall and national subsamples. Additional file 8 (docx): Subjective state changes (national subsamples). Additional file 9 (docx): Heart rate variability descriptive data (overall sample). Additional file 10 (docx): HRV analysis (national subsamples). [file 6673190.f1.zip › 6673190.f1/Additional file 10 (1).docx]

**HRV analysis for national subsamples**

Changes of HRV indicators followed a similar trend in the Chinese and German subsample (SI Figure 3). lnHF and RMSSD showed a tendency to decrease during Qi gong compared to rest whereas lnVLF, SDNN and HR showed an increase during Qi Gong (S8 – S9 Table). No significant differences between the resting periods were found (all p > .10). The overall effects for lnHF, F_(1.3, 22.5)_ = 25.75, p < .001, partial η² = 0.6, lnVLF, F_(1.5, 25.8)_ = 36.57, p < .001, partial η² = 0.68, RMSSD, F_(1.2, 20.7)_ = 14.81, p = .001, partial η² = 0.47, SDNN, F_(1.3, 23.1)_ = 5.67, p = .018, partial η² = 0.25 and HR, F_(1.4, 23.2)_ = 214.83, p < .001, partial η² = 0.93 were significant and more pronounced in the Chinese sample whereas in the German sample the overall effect was only significant for HR, F_(1.6, 21.9)_ = 177.56, p < .001, partial η² = 0.93 and lnVLF, F_(2, 25.9)_ = 5.34, p = .012, partial η² = 0.29 and lnHf (p = .103), RMSSD (p = .106) showed a trend for significance.

No sample showed an overall difference in coherence (p > .10). The effect for SPcoherence was only found in the Chinese sample F_(1.3, 22.3)_ = 6.37, p = .013, partial η² = 0.27, whereas in the German sample no overall difference between conditions was found (p = .57).
