# Supplementary material for: Relaxation or Regulation: The Acute Effect of Mind-Body Exercise on Heart Rate Variability and Subjective State in Experienced Qi Gong Practitioners
Source: Evid Based Complement Alternat Med. 2021 Jun 8;2021:6673190. doi: 10.1155/2021/6673190 (PMC8208883; doi:10.1155/2021/6673190)
Supplement: Supplementary Materials — Additional files. Additional file 1 (docx): National subsample characteristics. Additional file 2 (docx): Subjective state items in English, Chinese, and German. Additional file 3 (docx): Generation and factor-scale analysis of Qi belief items. Additional file 4 (docx): Belief items in English, Chinese, and German. Additional file 5 (docx): Rotated factor loadings, Eigenvalue, and Cronbach's Alpha of all belief items. Additional file 6 (docx): Rotated factor loadings, Eigenvalue, and Cronbach's Alpha of selected belief items. Additional file 7 (docx): Changes in subjective state over experiment in overall and national subsamples. Additional file 8 (docx): Subjective state changes (national subsamples). Additional file 9 (docx): Heart rate variability descriptive data (overall sample). Additional file 10 (docx): HRV analysis (national subsamples). [file 6673190.f1.zip › 6673190.f1/Additional file 2.docx]

|  | **EN** | **CN** | **GER** |
| --- | --- | --- | --- |
| 1 | I feel alive and vital | 我感觉很有活力和生命力 | fühle ich mich vital und lebendig |
| 2 | I have energy and spirit | 我很有能量、动力和热情 | habe ich Energie und Elan |
| 3 | I feel calm | 我内心安定 | fühle ich mich ruhig |
| 4 | I feel pleasure in my body | 我身体感觉舒适 | habe ich ein angenehmes Körpergefühl |
| 5 | My attention is focused | 我的注意力集中 | ist meine Aufmerksamkeit fokussiert |
| 6 | I can sense my own body | 我能感觉到自己的身体 | spüre ich meinen Körper |
|  | My body and mind are in harmony | 我身心合一 | sind mein Körper und Geist in Einklang |
|  | My body feels activated | 我身体感觉活力 | fühlt sich mein Körper aktiviert an |
|  | I feel energy flowing through my body | 我感觉身体里流淌着能量 | habe ich das Gefühl von Energie durchströmt zu werden |
|  | I can feel the Qi | 我能感觉到气 | kann ich das Qi spüren |
