# Supplementary material for: Relaxation or Regulation: The Acute Effect of Mind-Body Exercise on Heart Rate Variability and Subjective State in Experienced Qi Gong Practitioners
Source: Evid Based Complement Alternat Med. 2021 Jun 8;2021:6673190. doi: 10.1155/2021/6673190 (PMC8208883; doi:10.1155/2021/6673190)
Supplement: Supplementary Materials — Additional files. Additional file 1 (docx): National subsample characteristics. Additional file 2 (docx): Subjective state items in English, Chinese, and German. Additional file 3 (docx): Generation and factor-scale analysis of Qi belief items. Additional file 4 (docx): Belief items in English, Chinese, and German. Additional file 5 (docx): Rotated factor loadings, Eigenvalue, and Cronbach's Alpha of all belief items. Additional file 6 (docx): Rotated factor loadings, Eigenvalue, and Cronbach's Alpha of selected belief items. Additional file 7 (docx): Changes in subjective state over experiment in overall and national subsamples. Additional file 8 (docx): Subjective state changes (national subsamples). Additional file 9 (docx): Heart rate variability descriptive data (overall sample). Additional file 10 (docx): HRV analysis (national subsamples). [file 6673190.f1.zip › 6673190.f1/Additional file 3.docx]

**Qi Gong specific beliefs: Scale generation**

To assess Qi Gong specific beliefs in the two national samples 9 items were derived from discussions of the first author with various Qi Gong teachers based on public available training materials. In order to explore the structure of Qi Gong specific belief items we conducted a principal axis factor and reliability analysis on the nine belief items. We expected statistical dependency so an oblique rotation (direct oblimin) was used. An initial principal axis factor analysis on the nine items was run that yielded a three factor solution with Eigenvalues > 1 and together explained 62.5% of the variance. The Kaiser-Meyer-Olkin measure verified the adequacy of the sample size for the analysis, KM = 0.66 (1). The screeplot was ambiguous and showed inflexions that would justify a one-,two-, and three-factor solution. Reliability analysis for Factor 1 (Item 1,2,5,8) yielded a Cronbach's alpha α = .27. Item 8 ("Qi Gong mainly serves the purpose of disease prevention") was the only item whose exclusion indicated a reliability improvement. Omitting item 8 increased reliability of factor 1 to a Cronbach's alpha of α = .71. Factor 2 yielded a Cronbach's alpha of α = .65. Item 9 ("Qi Gong can accelerate the process of recovery from a disease") was the only item to comprise a single factor and was therefore omitted. SI Table 10 shows the rotated factor loadings, Eigenvalues and, explained % of variance and α of the original items.

We repeated the principal factor analysis with oblique rotation after omission of item 8 and 9 which yielded a two-factor solution with Eigenvalues >1 that together explained 58.2% of the variance. All items had their highest loading on the same factor as in the prior analysis (SI Table 11). Factor 1 comprised the items 3,4,6 (reversed) and 7 which focus on the nature of Qi and it's accessability to scientific investigation. Factor 2 comprised items 1,2 and 5 (reversed) with a focus on the existence of Qi. Due to the original order of the items the factors were described in a different order in the orginal paper. Factor one was "Belief in Qi" (Item 1: "During Qi Gong I can feel my Qi"; Item 2: "I don't believe in the existence of Qi (r: to be recoded)"; Item 3: "There is something like Qi, however science has yet to succeed in measuring it"). Factor two was "Belief in the scientific investigatability of Qi" (Item1: "There is a scientific explanation for Qi"; Item 2: "Qi is something that can't be explained by science (r: to be recoded)"; Item 3: "Qi is a sensation which emerges during the alignment of movement, attention and breath"; Item 4: "Qi Gong as intervention is also capable of curing serious disease, such as cancer"). The items were translated into Chinese and German using a backward-forward approach in a similar manner as the subjective state items (SI Table 2).
