# Supplementary material for: Relaxation or Regulation: The Acute Effect of Mind-Body Exercise on Heart Rate Variability and Subjective State in Experienced Qi Gong Practitioners
Source: Evid Based Complement Alternat Med. 2021 Jun 8;2021:6673190. doi: 10.1155/2021/6673190 (PMC8208883; doi:10.1155/2021/6673190)
Supplement: Supplementary Materials — Additional files. Additional file 1 (docx): National subsample characteristics. Additional file 2 (docx): Subjective state items in English, Chinese, and German. Additional file 3 (docx): Generation and factor-scale analysis of Qi belief items. Additional file 4 (docx): Belief items in English, Chinese, and German. Additional file 5 (docx): Rotated factor loadings, Eigenvalue, and Cronbach's Alpha of all belief items. Additional file 6 (docx): Rotated factor loadings, Eigenvalue, and Cronbach's Alpha of selected belief items. Additional file 7 (docx): Changes in subjective state over experiment in overall and national subsamples. Additional file 8 (docx): Subjective state changes (national subsamples). Additional file 9 (docx): Heart rate variability descriptive data (overall sample). Additional file 10 (docx): HRV analysis (national subsamples). [file 6673190.f1.zip › 6673190.f1/Additional file 4.docx]

|  | **EN** | **CN** | **GER** |
| --- | --- | --- | --- |
| **Belief in Qi** | | | |
| 1 | During Qi Gong I can feel my Qi | 在练健身气功的时候我能感觉到气 | Während Qi Gong kann ich mein Qi spüren |
| 2 | I don't believe in the existence of Qi (r) | 我不相信气是存在的 | Ich glaube nicht, das es so etwas wie Qi gibt |
| 3 | There is something like Qi, however science has yet to succeed in measuring it | 气是存在的，只是现在的科学还无法测量它 | Es gibt so etwas wie Qi, die Wissenschaft konnte es nur noch nicht messen |
| **Belief in the scientific investigatability of Qi** | | | |
| 1 | There is a scientific explanation for Qi | 气这种现象是有可以用科学解释的 | Für Qi gibt es eine naturwissenschaftliche Erklärung |
| 2 | Qi is something that can't be explained by science (r) | 气这种现象是科学无法解释的 | Qi ist etwas, dass sich nicht wissenschaftlich erklären lässt |
| 3 | Qi is a sensation which emerges during the alignment of movement, attention and breath | 气是一种感知，气随着运动、注意力和呼吸的一体化而形成的整体 | Qi ist eine Wahrnehmung, welche bei der Integration Bewegung, Aufmerksamkeit und Atem in eine Einheit entsteht |
| 4 | Qi Gong as intervention is also capable of curing serious disease, such as cancer | 健身气功可以作为一个独立的干预，包括严重的疾病，如癌症治疗 | Qi Gong als eigenständige Intervention kann auch schwere Krankheiten, wie z.B. Krebs heilen |
| **Excluded Items** | | | |
| 1 | Qi Gong mainly serves the purpose of disease prevention | 健身气功，主要用于疾病的预防 | Qi Gong dient vor allem der Vorbeugung von Krankheiten |
| 2 | Qi Gong can accelerate the process of recovery from a disease | 健身气功，主要用于疾病的预防 | Qi Gong kann den Heilungsprozess nach einer Krankheit beschleunigen |
