# Supplementary material for: Relaxation or Regulation: The Acute Effect of Mind-Body Exercise on Heart Rate Variability and Subjective State in Experienced Qi Gong Practitioners
Source: Evid Based Complement Alternat Med. 2021 Jun 8;2021:6673190. doi: 10.1155/2021/6673190 (PMC8208883; doi:10.1155/2021/6673190)
Supplement: Supplementary Materials — Additional files. Additional file 1 (docx): National subsample characteristics. Additional file 2 (docx): Subjective state items in English, Chinese, and German. Additional file 3 (docx): Generation and factor-scale analysis of Qi belief items. Additional file 4 (docx): Belief items in English, Chinese, and German. Additional file 5 (docx): Rotated factor loadings, Eigenvalue, and Cronbach's Alpha of all belief items. Additional file 6 (docx): Rotated factor loadings, Eigenvalue, and Cronbach's Alpha of selected belief items. Additional file 7 (docx): Changes in subjective state over experiment in overall and national subsamples. Additional file 8 (docx): Subjective state changes (national subsamples). Additional file 9 (docx): Heart rate variability descriptive data (overall sample). Additional file 10 (docx): HRV analysis (national subsamples). [file 6673190.f1.zip › 6673190.f1/Additional file 6.docx]

|  |  |  |  |  |
| --- | --- | --- | --- | --- |
|  | Item | Factor 1 | Factor 2 |  |
| 5 | I don't believe in the existence of Qi (r) | **-0.98** | -0.15 |  |
| 2 | During Qi Gong, I can feel my Qi | **-0.58** | 0.21 |  |
| 1 | There is something like Qi, however science has yet to succeed in measuring it | **-0.43** | 0.12 |  |
| 4 | There is a scientific explanation for Qi | 0.03 | **0.94** |  |
| 7 | Qi Gong as intervention is also capable of curing serious disease, such as cancer | 0.04 | **0.55** |  |
| 6 | Qi is something that can't be explained by science (r) | -0.26 | **0.4** |  |
| 3 | Qi is a sensation which emerges during the alignment of movement, attention and breath | -0.05 | **0.35** |  |
|  | Eigenvalues | 1.02 | 3.06 |  |
|  | % of variance | 14.56 | 43.67 |  |
|  | Cronbach's α | .71 | .65 |  |
