# Supplementary material for: Relaxation or Regulation: The Acute Effect of Mind-Body Exercise on Heart Rate Variability and Subjective State in Experienced Qi Gong Practitioners
Source: Evid Based Complement Alternat Med. 2021 Jun 8;2021:6673190. doi: 10.1155/2021/6673190 (PMC8208883; doi:10.1155/2021/6673190)
Supplement: Supplementary Materials — Additional files. Additional file 1 (docx): National subsample characteristics. Additional file 2 (docx): Subjective state items in English, Chinese, and German. Additional file 3 (docx): Generation and factor-scale analysis of Qi belief items. Additional file 4 (docx): Belief items in English, Chinese, and German. Additional file 5 (docx): Rotated factor loadings, Eigenvalue, and Cronbach's Alpha of all belief items. Additional file 6 (docx): Rotated factor loadings, Eigenvalue, and Cronbach's Alpha of selected belief items. Additional file 7 (docx): Changes in subjective state over experiment in overall and national subsamples. Additional file 8 (docx): Subjective state changes (national subsamples). Additional file 9 (docx): Heart rate variability descriptive data (overall sample). Additional file 10 (docx): HRV analysis (national subsamples). [file 6673190.f1.zip › 6673190.f1/Additional file 7 (1).docx]

| **Overall sample** (CN/GER)  n=39 (n=19/n=20) | Rest 0  (t0-t1) | Qi Gong  (t0-t3) | Qi Gong  t1-t3 | Rest 1  (t3-t4) |
| --- | --- | --- | --- | --- |
| Subjective Vitality | **O** (O/O)  .223  (>.99/.095) | **↗** (O/↗)  <.001 (.281/.004) | **↗** (↗/↗)  <.001 (.008/<.001) | **↘**(O/O)  .018  (>.99/.051) |
|  |  |  |  |  |
| Calmness | **↗** (O/O)  .002 (.065/.115) | **↗** (O/↗)  .001  (.065/.034) | **O** (O/O)  >.99 (>.99/>.99) | **O** (O/O)  >.99  (>.99/.493) |
| Pleasant body sensation | **O** (O/O)  .163  (.232/>.99) | **↗**(O/↗)  .002  (.896/.001) | **O** (O/↗)  .052  (>.99/.015) | **O** (O/O)  >.99 (>.99/>.99) |
| Focused attention | **O** (O/O)  .864  (.073/>.99) | **↗**(↗/↗)  <.001 (.041/.025) | **↗** (O/↗)  .004  (.419/.039) | **O** (O/O)  >.99  (.962/>.99) |
| Body awareness | **O** (O/O)  >.99  (>.99/>.99) | **↗** (↗/↗)  <.001 (.034/.031) | **↗**(O/↗)  <.001 (.083/.008) | **O** (O/O)  >.99  (.15/>.99) |
| Perceived body activation | **O** (O/O)  >.99  (>.99/.486) | **↗** (↗/↗)  <.001 (.014/.001) | **↗** (↗/↗)  <.001 (.014/<.001) | **O** (O/O)  .051  (.349/.662) |
|  |  |  |  |  |
| Sensation of Qi | **O** (O/O)  .118 (.419/.897) | **↗**(↗/↗)  <.001 (.001/<.001) | **↗** (↗/↗)  <.001 (.001/.001) | **↘** (O/O)  .003 (.082/.099) |
