# Supplementary material for: Relaxation or Regulation: The Acute Effect of Mind-Body Exercise on Heart Rate Variability and Subjective State in Experienced Qi Gong Practitioners
Source: Evid Based Complement Alternat Med. 2021 Jun 8;2021:6673190. doi: 10.1155/2021/6673190 (PMC8208883; doi:10.1155/2021/6673190)
Supplement: Supplementary Materials — Additional files. Additional file 1 (docx): National subsample characteristics. Additional file 2 (docx): Subjective state items in English, Chinese, and German. Additional file 3 (docx): Generation and factor-scale analysis of Qi belief items. Additional file 4 (docx): Belief items in English, Chinese, and German. Additional file 5 (docx): Rotated factor loadings, Eigenvalue, and Cronbach's Alpha of all belief items. Additional file 6 (docx): Rotated factor loadings, Eigenvalue, and Cronbach's Alpha of selected belief items. Additional file 7 (docx): Changes in subjective state over experiment in overall and national subsamples. Additional file 8 (docx): Subjective state changes (national subsamples). Additional file 9 (docx): Heart rate variability descriptive data (overall sample). Additional file 10 (docx): HRV analysis (national subsamples). [file 6673190.f1.zip › 6673190.f1/Additional file 8 (1).docx]

**Subjective state changes: Analysis in the Chinese and the German subsample**

The general trend of increase in subjective vitality following Qi Gong compared to no changes/decrease during the resting conditions was similar and significant in both national subsamples (S3 Table). The German sample showed a tendency of decrease in SV during the resting periods (t0-t1; p = .095; t3-t4; p = .051) whereas the Chinese sample reached a peak in SV following the first Qi Gong exercise (t0-t2; p = .015) with a decrease afterwards (t0-t3; p = .281).

For all subjective state variables except pleasant body sensation the two subsamples showed similar changes as in the overall sample (S3 Table). Focused attention, body awareness and perceived body activation showed no change over initial rest and significant increase during one or both Qi Gong exercise compared to initial baseline (p <.05). Calmness showed a trend for a significant increase during rest in the Chinese (t0-t1; p = .065), less so in the German (t0-t1; p =.115) subsample and remained on this level. In the Chinese sample pleasant body sensation showed a trend for a significant overall difference over the course of the experiment (p = .101). In the German sample pleasant body sensations were significantly higher following Qi Gong (t0-t3; p = .001; t1-t3; p = .015) and did not change over the second resting period (p >.10). All changes and the respective p-values are reported in S3 Table.
