# Supplementary material for: Relaxation or Regulation: The Acute Effect of Mind-Body Exercise on Heart Rate Variability and Subjective State in Experienced Qi Gong Practitioners
Source: Evid Based Complement Alternat Med. 2021 Jun 8;2021:6673190. doi: 10.1155/2021/6673190 (PMC8208883; doi:10.1155/2021/6673190)
Supplement: Supplementary Materials — Additional files. Additional file 1 (docx): National subsample characteristics. Additional file 2 (docx): Subjective state items in English, Chinese, and German. Additional file 3 (docx): Generation and factor-scale analysis of Qi belief items. Additional file 4 (docx): Belief items in English, Chinese, and German. Additional file 5 (docx): Rotated factor loadings, Eigenvalue, and Cronbach's Alpha of all belief items. Additional file 6 (docx): Rotated factor loadings, Eigenvalue, and Cronbach's Alpha of selected belief items. Additional file 7 (docx): Changes in subjective state over experiment in overall and national subsamples. Additional file 8 (docx): Subjective state changes (national subsamples). Additional file 9 (docx): Heart rate variability descriptive data (overall sample). Additional file 10 (docx): HRV analysis (national subsamples). [file 6673190.f1.zip › 6673190.f1/Additional file 9 (1).docx]

| Overall sample | RS_0  (n=40) | *SD* | Qi_1  (n=36) | *SD* | Qi_2  (n=34) | *SD* | RS_1  (n=37) | *SD* |
| --- | --- | --- | --- | --- | --- | --- | --- | --- |
| HF | 1093 | (1820) | 212 | (262) | 214 | (283) | 966 | (1735) |
| LF | 1108 | (1460) | 1890 | (2008) | 1943 | (2038) | 877 | (1336) |
| VLF | 730 | (660) | 2311 | (2042) | 2522 | (1978) | 966 | (1204) |
| lnHF | 5.83 | (1.69) | 4.74 | (1.22) | 4.78 | (1.12) | 5.72 | (1.66) |
| lnLF | 6.23 | (1.35) | 6.96 | (1.26) | 7.01 | (1.29) | 6.01 | (1.27) |
| lnVLF | 6.23 | (0.9) | 7.35 | (0.98) | 7.47 | (0.98) | 6.31 | (1.07) |
| HF (n.u.) | 41.62 | (23.33) | 11.94 | (9.7) | 11.38 | (8.88) | 44.52 | (25.13) |
| LF (n.u.) | 58.3 | (23.39) | 88.02 | (9.77) | 88.57 | (8.92) | 55.32 | (25.23) |
| Coherence | 0.43 | (0.35) | 0.52 | (0.46) | 0.48 | (0.36) | 0.32 | (0.26) |
| Coherence SP | 0.87 | (0.7) | 1.51 | (1.87) | 1.33 | (0.74) | 0.65 | (0.48) |
|  |  |  |  |  |  |  |  |  |
| RMSSD | 45.19 | (35.81) | 22.85 | (10.16) | 23.38 | (10.3) | 45.72 | (41.28) |
| SDNN | 51.45 | (27.24) | 64.3 | (24.69) | 65.83 | (23.4) | 51.89 | (30.48) |
| SD1 | 32.02 | (25.37) | 16.17 | (7.19) | 16.54 | (7.29) | 32.38 | (29.24) |
| SD2 | 64.11 | (31.53) | 89.28 | (34.5) | 91.45 | (32.76) | 64.27 | (34.7) |
|  |  |  |  |  |  |  |  |  |
| HR | 66.76 | (9.11) | 90.02 | (10.03) | 91.37 | (9.17) | 65.98 | (8.7) |
| Total Power (ms²) | 2934 | (3110) | 4414 | (3515) | 4681 | (3498) | 2814 | (3258) |
| Power Peak  (0.04Hz-0.4 Hz) | 0.13 | (0.07) | 0.06 | (0.02) | 0.06 | (0.02) | 0.15 | (0.09) |
